# Endoscopic recurrence of Crohn’s disease following laparoscopic versus robotic ileocolic resection

**DOI:** 10.1007/s10151-026-03367-9

**Published:** 2026-05-26

**Authors:** Marissa C. Kuo, Samuel A. Younan, Phillip J. Williams, Hanjoo Lee, Baldeep Pabla, Sara Horst, Alexander T. Hawkins, Aimal Khan

**Affiliations:** 1 Vanderbilt University Medical Center, 1161 21st Avenue South, Nashville, TN 37232-2730 USA; 2 Division of Colon and Rectal Surgery, Harbor-UCLA Medical Center, Torrance, CA USA; 3 Division of Gastroenterology, Hepatology, and Nutrition, Vanderbilt University Medical Center, Nashville, TN USA; 4 Division of Colon and Rectal Surgery, Vanderbilt University Medical Center, Nashville, TN USA

**Keywords:** Crohn’s disease, Endoscopic recurrence, Laparoscopic surgery, Robotic surgery, Iileocolic resection, Rutgeerts

## Abstract

**Background:**

Robotic approach is increasingly being used for surgery in Crohn’s disease. Robotic ileocolic resection (ICR) for Crohn’s disease (CD) is comparable to laparoscopic ICR for short term outcomes; however, no studies have compared longer-term recurrence rates between these approaches.

**Objective:**

The objective was to compare longer-term recurrence rates between these approaches.

**Methods:**

We performed a retrospective cohort study using a deidentified single institution research database containing data extracted from the electronic health record. We included all patients with CD who underwent minimally invasive ICR between 2017 and 2024. The primary outcome was endoscopic recurrence within 18 months postoperatively, defined as Rutgeerts score ≥ i2. The time-to-event analysis was performed to compare time to endoscopic recurrence between laparoscopic versus robotic ICR.

**Results:**

A total of 164 patients were included in study, with a predominant majority prescribed an advanced medical therapy postoperatively (93.9%). Within 18 months postoperatively, 22/164 (13.4%) patients experienced endoscopic recurrence: 16/127 (12.5%) in the laparoscopic cohort and 6/37 (16.2%) in the robotic cohort. In the time-to-event analysis, there was no statistically detectable difference in Kaplan–Meier curves compared between laparoscopic and robotic ICR (log rank *p* = 0.115). In multivariable Cox proportional hazards models adjusted for smoking history and history of ICR, there was no statistically detectable difference in endoscopic recurrence rates within 18 months postoperatively compared between laparoscopic and robotic ICR (HR 1.12, 95% CI 0.341–3.74, *p* = 0.842).

**Conclusions:**

In this retrospective cohort, no statistically detectable differences in endoscopic recurrence between approaches was observed; however, conclusions are limited by small sample size, low event rates, and widespread postoperative biologic use and should be interpreted as exploratory only..

## Introduction

Crohn’s disease (CD) is a chronic relapsing inflammatory bowel disease that frequently affects the terminal ileum [1]. Upward of 80% of patients with CD will require surgical intervention at some point during their lifetime [2, 3]. The goal of surgery in the setting of CD is to relieve symptomatic disease and improve quality of life. Advances in medical therapy for CD have been made in recent decades. However, disease recurrence is still common, and many patients who undergo surgery for CD will require multiple surgeries throughout their lifetime [4]. Endoscopic disease recurrence has been shown to be a key predictor of subsequent clinical and surgical disease recurrence [5].

Minimally invasive techniques for CD are well established and are incorporated into the guidelines of the American Society of Colon and Rectal Surgeons and the European Crohn’s and Colitis Organization guidelines [6, 7]. Laparoscopic approach has been shown to be associated with reduced postoperative pain and shorter hospital stays compared with open approach [8–11]. In recent years, robotic approach has emerged as an alternative to laparoscopy, offering potential advantages such as enhanced dexterity and greater precision [12, 13]. Most current robotic platforms however, lack haptic feedback mechanisms which may limit a surgeons ability to assess for macroscopic disease by palpation. The ability to obtain macroscopically disease-free margin is recommended by the American Society of Colon and Rectal Surgeons in order to mitigate disease recurrence risk while conserving bowel length [14]. Numerous small studies demonstrate the feasibility and safety of robotic surgery broadly for colorectal disease and for inflammatory bowel disease compared with the laparoscopic approach [15, 16]. However, the role of robotic surgery in the treatment of CD remains poorly defined, and there is a paucity of data on its impact on disease recurrence at the anastomotic site.

To address this knowledge gap, we sought to describe and explore endoscopic recurrence patterns following laparoscopic versus robotic ileocolic resection (ICR) in patients with Crohn’s disease. Our objective to explore whether postoperative recurrence rates are impacted by evaluate platform-related technical factors and real-world practice patterns during the adoption of the robotic approach.

## Methods

### Study design, setting, and data sources

We performed a retrospective cohort study using the Vanderbilt Institute for Clinical and Translational Research Synthetic Derivative (SD), a deidentified database of more than 3.9 million longitudinal patient electronic health records from Vanderbilt University Medical Center spanning over 15 years. SD records are deidentified using electronic scrubbing techniques to remove identifiers while maintaining semantic integrity. The SD is compliant with HIPAA Safe Harbor standards and is structured according to the Observational Medical Outcomes Partnership common data model. The SD is updated every 6 months, typically with a 6-week delay, and at the time of our study, data was available up through 31 August 2024. SD Discover, a self-service web tool, was used for cohort selection and data extraction. This study was approved by the Vanderbilt University Medical Center Institutional Review Board, and the need for informed consent was waived owing to the deidentified nature of the database in use (institutional review board (IRB) no. 242068).

### Study population and data collection

The SD was first queried to identify all patient records containing an ICD9 or ICD10 diagnosis code of regional enteritis or Crohn’s disease respectively (ICD-9 555.0, 555.1, 555.2, 555.9, ICD10 K50.0, 50.1, 50.8, 50.9) and at least one patient encounter associated with CPT procedure code 44205 for laparoscopic partial colectomy with the removal of the terminal ileum and ileocolostomy. As there are no CPT procedure codes specific for robotic partial colectomy, all minimally invasive ICRs are coded under this single CPT code at our institution. There were no duplicate records identified (i.e., no patients had undergone multiple ICRs within the study period). We then excluded all patient records whose first patient encounter associated with CPT procedure code 44205 was prior to 1 January 2017. This was done because there were no surgeons at our institution who were performing robotic surgery for CD prior to the year 2017. The remaining records were then manually reviewed, and additional records were excluded if there were no colonoscopy reports available in SD evaluating the ileocolic anastomosis postoperatively. Records were also excluded if an end ileostomy was created at the time of ICR or if the operative note described conversion from a minimally invasive to an open approach during ICR. Clinical information was obtained via the manual review of SD records. Each record was reviewed by author MCK using a structured chart abstraction form. Data for review included patient clinicopathologic covariates, operative notes, prescribed medications, and endoscopy procedure reports. All data were stored using a secured REDCap instrument [17, 18].

### Exposure and outcome

The primary predictor variable was the operative approach for ICR: laparoscopic or robotic. The operative approach was identified via the manual review of the operative report. Our primary outcome was endoscopic recurrence at the ileocolic anastomosis within 18 months of ICR. Our follow-up period of 18 months was selected on the basis of precedent in literature, namely the SuPREMe-CD trial, evaluating endoscopic recurrence after Kono-S anastomosis [19]. Endoscopic recurrence was defined as Rutgeerts score ≥ i2, as determined and documented by the endoscopist [20]. Scores were obtained via the manual review of endoscopy procedure reports via SD records. If a record had multiple postoperative colonoscopies documenting Rutgeerts score ≥ i2 within the study period, the date of the first colonoscopy showing recurrence was recorded as the date of recurrence. Procedures in this study were performed by six colorectal surgeons, all of whom had established experience with laparoscopic ileocolic resection; robotic cases were performed during the institutional adoption period of the robotic platform.

### Statistical analysis

Continuous variables (age and body mass index) were evaluated for distribution using histogram plots and were summarized using median and interquartile range. Categorical variables were evaluated using counts and percentages. The primary outcome of endoscopic recurrence at the ileocolic anastomosis within 18 months was evaluated using Kaplan–Meier failure plots with log rank test to compare endoscopic recurrence rates between the laparoscopic and robotic cohorts. Multivariable Cox proportional hazard models, adjusted for smoking history and history of ICR, were used to evaluate recurrence rates between the laparoscopic and robotic approach. The postoperative use of biologic medication (yes versus no) was excluded from the multivariable Cox model as more than 90% of patients in both cohorts were documented as having been prescribed a biologic postoperatively. Additionally, the variable demonstrated perfect colinearity with the exposure variable (Pearson *r* = 1.0). The number of additional covariates adjusted for in the model was limited by the number of outcomes in each cohort. Statistical significance criterion of *p* ≤ 0.05 was used. All data analyses were performed using Stata Statistical Software version 18.0 (StataCorps, College Station, TX, USA).

### Sensitivity analysis

A sensitivity analysis was conducted to test the robustness of the results. First, we wished to also evaluate endoscopic recurrence rates using the total follow-up period available for both cohorts (as opposed to recurrence within 18 months) given the variable follow-up available per record. Conducting this additional analysis using person-years can help offset the impact of the low number of robotic resections. Unadjusted and adjusted incidence rate ratios were calculated for endoscopic recurrence per person-year. Total person-year follow-up was calculated for the laparoscopic and robotic cohorts, which were then used to calculate raw incidence rates by operative approach. This allowed for the calculation of an unadjusted incidence rate ratio for endoscopic recurrence between the robotic and laparoscopic approach. An adjusted incidence rate ratio was then calculated using multivariable Poisson regression models adjusted for smoking and history of ICR. Postoperative biologic medication prescribed within 6 months of surgery was omitted from the model, for the reasons explained above.

## Results

### Study cohort and characteristics

**Fig. 1:**
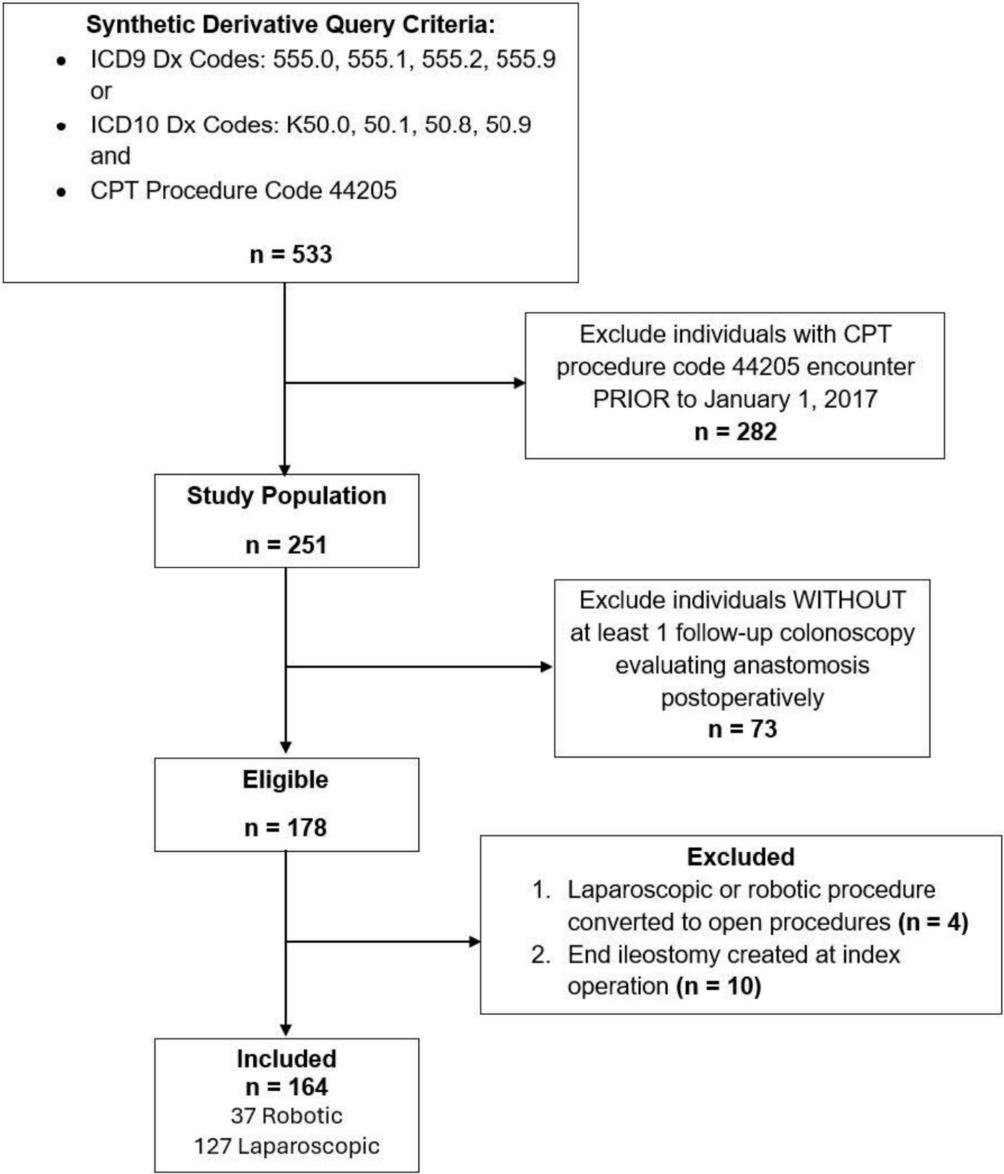
Cohort creation

A total of 251 SD records were reviewed; 73 records were excluded as they did not contain documentation of any postoperative endoscopic evaluation of the ileocolic anastomosis. An additional four patients had undergone minimally invasive conversion to open ICR and were also excluded. Finally, ten records indicated in the operative report that the patient underwent end ileostomy at the time of ICR, and these records were excluded as well. The cohort creation is depicted in Fig. 1.

There were 164 records remaining in the analytic cohort, including 127 (77.5%) laparoscopic and 37 (23.5%) robotic ileocolic resections. The majority of patients were female (55.5%). The cohort median age was 32 years with a median body mass index (BMI) of 24.1 kg/m^2^. The majority of patients had no history of prior ileocolic resection (85.9%) and had not been hospitalized within the 3 months preoperatively (69.3%). A minority of patients were prescribed intravenous (IV) or per os (PO) corticosteroids in the three months preoperatively (28.0%). Almost all patients in both cohorts were prescribed an advanced therapy within the 6 months postoperatively (94.5% in laparoscopic cohort, 91.9% in robotic cohort). Fewer patients in the robotic cohort underwent diverting loop ileostomy compared with the laparoscopic cohort (10.8% versus 16.5%, respectively). In total, 33% of patients were former or current smokers. Otherwise, covariates were relatively balanced between the two cohorts. Complete patient demographics and characteristics are described in Table 1.

**Table 1:** Patient demographics

Characteristic	Median (Q1, Q3), *n* (%)		
	Laparoscopic *n* = 127	Robotic *n* = 37	Total *n* = 164
Age (years)	32 (22, 44)	33 (27, 53)	32 (22, 45.5)
BMI	23.9 (20.8, 27.8)	25.3 (21.3, 29.4)	24.1 (20.9, 28.4)
Sex			
Female	73 (57.5%)	18 (48.6%)	91 (55.5%)
Male	54 (42.5%)	19 (51.4%)	73 (44.5%)
*Smoking history*			
Current	15 (11.8%)	3 (8.1%)	18 (11.0%)
Former	27 (21.3%)	10 (27.0%)	37 (22.6%)
Never	85 (66.9%)	24 (64.9%)	109 (66.5%)
Hospitalizations within 3 months preoperatively			
Zero	87 (69.0%)	26 (70.3%)	113 (69.3%)
One	31 (24.6%)	9 (24.3%)	40 (24.5%)
Two or more	8 (6.3%)	2 (5.4%)	10 (6.1%)
History of ICR			
None	109 (86.5%)	31 (83.8%)	140 (85.9%)
One	16 (12.7%)	5 (13.5%)	21 (12.9%)
Two or more	1 (0.8%)	1 (2.7%)	2 (1.2%)
Underwent loop ileostomy at index operation			
Yes	21 (16.5%)	4 (10.8%)	25 (15.2%)
No	106 (83.5%)	33 (89.2%)	139 (84.8%)
Preoperative corticosteroids (IV or PO)^			
Yes	37 (29.1%)	9 (24.3%)	46 (28.0%)
No	90 (70.9%)	28 (75.7%)	118 (72.0%)
Preoperative biologic medication*			
Yes	96 (75.6%)	25 (67.6%)	121 (73.7%)
No	31 (24.4%)	12 (32.4%)	43 (26.2%)
Postoperative biologic medication*			
Yes	120 (94.5%)	34 (91.9%)	154 (93.9%)
No	7 (5.5%)	3 (8.1%)	10 (6.1%)

Biologics medications included: adalimumab, certolizumab, infliximab, golimumab, natalizumab, risankizumab, tacrolimus, tofacitinib, upadacitinib, ustekinomab, vedolizumab

*BMI* body mass index (m/kg^2^), *ICR* ileocolic resection, *IV* intravenous, *PO* per os

^Defined as within 3 months preoperatively

*Defined as within 6 months pre- or postoperatively

**Defined as Rutgeerts score ≥ i2

### Endoscopic recurrence within 18 months postoperatively

**Fig. 2:**
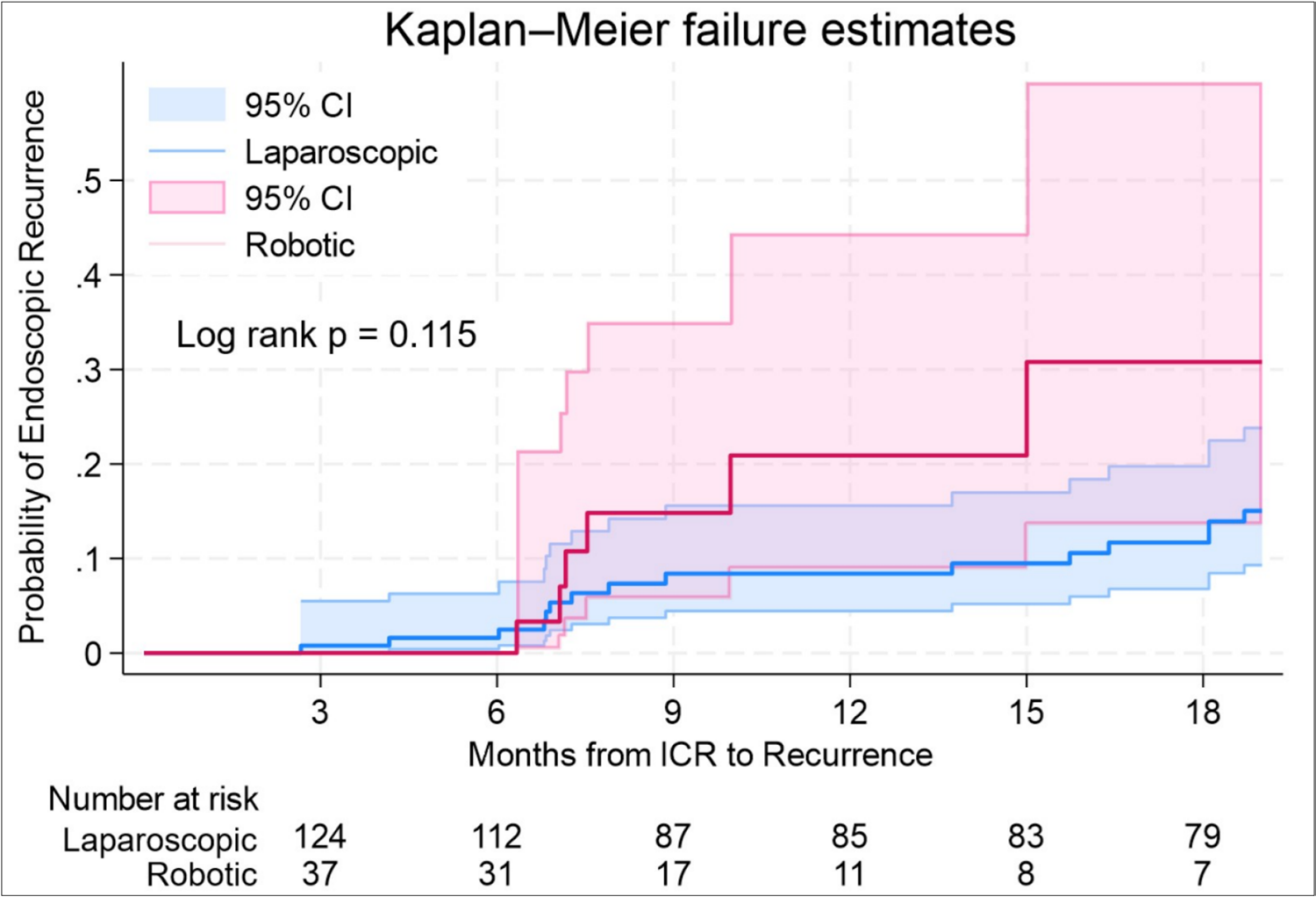
Kaplan–Meier curves showing endoscopic recurrence up to 18 months postoperatively following laparoscopic versus robotic ileocolic resection in patients with Crohn’s disease. Differences between groups were evaluated using the log-rank test (*p* = 0.115)

In this cohort study, median time from surgery to first endoscopy was 8.4 months (6.8, 10.1) in the laparoscopic cohort and 7.4 months (7.1, 9.9) in the robotic cohort. The overall rate of occurrence among the entire study sample was 13.4% (22 patients). A total of 16 out of 127 (12.5%) individuals who underwent laparoscopic ICR experienced endoscopic recurrence within 18 months postoperatively compared with 6 out of 37 (16.2%) individuals who underwent robotic ICR. Kaplan–Meier failure curves depicting the probability of recurrence by months since ICR are compared by operative approach, shown in Fig. 2.

There was no significant difference between the two approaches (log rank *p*-value = 0.155). In the multivariable Cox proportional hazards model adjusted for smoking history and history of ICR (no versus yes), there was no significant difference in endoscopic recurrence within 18 months of ICR between the laparoscopic and robotic approach, with an HR of 1.12 (95% CI 0.341–3.74, *p* = 0.842) (Table 2).

**Table 2:** Multivariable Cox proportional hazards regression model for factors associated with endoscopic recurrence within 18 months postoperatively

Variable	Reference	Comparison	HR (95% CI)	*p*-Value
Operative approach	Laparoscopic	Robotic	1.12 (0.34, 3.74)	0.842
Smoking history	Current	Former	0.304 (0.06, 1.60)	0.160
	Current	Never	2.03 (0.30, 13.7)	0.465
History of ICR	No	Yes	1.47 (0.39, 5.49)	0.569

*ICR* Ileocolic resection

### Sensitivity analysis

Total follow-up time available for each of the laparoscopic and robotic groups was calculated at 104,233 and 14,154 days respectively. A total of 27 patients in the laparoscopic cohort experienced recurrence during the entire study period, yielding an unadjusted incidence rate of 0.0945 per person-year. In total, six patients in the robotic cohort experienced recurrence during the entire study period, yielding an unadjusted incidence rate of 0.155 per person-year. The unadjusted incidence rate ratio was calculated at 1.64 for endoscopic recurrence following robotic versus laparoscopic ICR per person-year. The multivariable Poisson regression model used to calculate an adjusted incidence rate ratio for endoscopic recurrence following robotic versus laparoscopic ICR per person-year was calculated at 1.57 (95% CI 0.64–3.83, *p* = 0.322).

## Discussion

In this retrospective cohort study, we found no statistically detectable difference in endoscopic recurrence within 18 months of laparoscopic versus robotic ICR. Undergoing surgery for CD typically requires patients to pause advanced therapies for several weeks, and frequent interruptions in medical treatment can negatively impact quality of life, potentially making surgery counterproductive [21]. Furthermore, surgery for CD often involves bowel resection, placing patients who require multiple operations over their lifetime at risk for short bowel syndrome [22, 23]. These considerations highlight the importance of identifying modifiable risk factors in surgical approach and technique that may influence recurrence. To our knowledge, this is the first study to attempt to characterize long-term endoscopic recurrence following robotic versus laparoscopic ICR.

While prior small studies have reported on hospital length of stay, 30-day readmission and complication rates, and rates of conversion to open procedure associated with robotic ICR, none to our knowledge have reported on longer-term recurrence rates [16, 24, 25]. One meta-analysis comparing the laparoscopic to the robotic approach for surgery for inflammatory bowel disease demonstrated the robotic approach was associated with lower postoperative complication rates and shorter length of stay compared with the laparoscopic approach; however, disease recurrence was not addressed [26]. This knowledge gap may reflect the relatively recent adoption of the robotic platform compared with the laparoscopic approach. However, as the use of robotic surgery for CD becomes more widespread, addressing these questions is increasingly important.

This study has several important limitations that must be emphasized upfront. First, the small number of endoscopic recurrence events (particularly in the robotic cohort) substantially limits statistical power and increases the likelihood of Type II error. As a result, the absence of a statistically significant difference should not be interpreted as evidence of equivalence between surgical approaches. Second, the nearly universal use of postoperative advanced medical therapy (93.9%) likely suppressed recurrence rates across both cohorts, creating a floor effect that may have obscured differences that were attributable to surgical technique alone. Factors that we were unable to account for, such as the use of postoperative metronidazole, may also impact recurrence rates. Additionally, the study period coincided with institutional adoption of robotic surgery for Crohn’s disease. Selection bias related to surgeon preference, patient complexity, and learning curve effects may therefore be present and could not be fully controlled for in this retrospective exploration. Lastly, the lower rate of diverting loop ileostomy in the robotic cohort may reflect unmeasured differences in case complexity, surgeon decision-making, or evolving practice patterns over time rather than an effect of surgical platform alone.

Endoscopic recurrence has been shown to precede clinical and surgical recurrence with reported rates after ICR ranging from 48% to 93% at 1 year postoperatively [20, 27]. Endoscopic recurrence in the SuPREMe-CD trial in the unexposed group was reported at 67.4% at 18 months, significantly higher than in our study [19]. This difference may reflect the setting in which the colonoscopies were performed. Procedures conducted as part of a randomized clinical trial by a dedicated research team may be more likely to yield more detailed data owing to required adherence to research regulations [28]. Accordingly, it is likely that our results may underestimate the true rate of anastomotic recurrence in both cohorts.

The likelihood of detecting endoscopic recurrence following ICR increases further out from surgical resection. One randomized controlled trial demonstrated that patients who underwent endoscopy at 6 months had lower recurrence rates at 18 months compared with patients who did not have endoscopy at 6 months [29]. Therefore, the timing of endoscopy postoperatively is important, as it appears to affect recurrence rates. The American Gastroenterological Association recommends endoscopic monitoring 6–12 months following surgical resection, as up to 90% of disease recurrences are detectable endoscopically within the first year if not on prophylactic therapy [30]. We did not directly adjust for timing of endoscopic evaluation in our study; however, the use of advanced therapies seems to stabilize endoscopic recurrence rates over time postoperatively. One study reported the endoscopic disease recurrence rates seems to be more stable over time in patients on Inflixumab, reporting 21% at 6 months and 22.4% at 18 month on anti-TNF therapy [31]. Given that 93.9% of our study cohort was on advanced therapy postoperatively, it is likely that our disease recurrence rates were likely not affected much by timing of endoscopy done for each patient, particularly as similar proportions of both cohorts were on advanced therapies (94.5% in laparoscopic cohort and 91.9% in robotic cohort). Additionally, median time till endoscopic evaluation was similar between our two cohorts (8.4 months in laparoscopic cohort versus 7.4 months in the robotic cohort). However, as noted above, it is likely that the near universal usage of postoperative advanced medical therapies in this study may obscure true postoperative recurrence rates, making these results difficult to interpret in isolation. Larger, multicenter studies must be performed to further explore these preliminary findings.

One of the common drawbacks of most robotic surgical platforms is the lack haptic feedback [32]. Whereas laparoscopic surgery allows for palpation of the bowel with tactile and proprioceptive feedback provided to the user via laparoscopic instruments, the robotic platform relies on artificial intelligence to simulate haptic modality [33]. For surgeons who operate on CD pathology, this may impact where a surgeon perceives negative disease margins to lie, which may ultimately impact disease recurrence rates. This impact may become more pronounced as more surgeons start using robotic platforms for CD bowel resections. While theoretical concerns have been raised regarding the lack of haptic feedback in robotic platforms and its potential impact on margin assessment, our study was not designed to isolate this effect. In practice, several surgeons in our cohort used laparoscopic instruments during robotic cases for bowel mobilization prior to docking, which could, in theory, allow for tactile assessment of the terminal ileum. As such, the robotic cohort reflects real-world hybrid practice rather than a purely platform-isolated comparison, limiting any mechanistic inference regarding haptic feedback and recurrence risk.

We elected to use the Rutgeerts scoring system in our definition of recurrence as our primary outcome [20]. While the Rutgeerts scoring system has never been formally validated, it has been used in numerous randomized clinical trials and is well known as a standard reporting score for anastomotic recurrence following ileocolic resection [19, 34]. While the system is relatively specific in its designations, there is still the possibility of user variability in scoring. This may have impacted our results, given that in our study, there was likely more than just one single endoscopist performing follow-up colonoscopies and assessing for recurrence (though given the deidentified nature of the SD, this is impossible to confirm).

In this retrospective, single-institution cohort, no statistically detectable difference in endoscopic recurrence within 18 months was observed following laparoscopic versus robotic ileocolic resection for Crohn’s disease. Given the limited sample size, low event rates, and high prevalence of postoperative biologic therapy, these findings should be interpreted as exploratory and hypothesis-generating. Larger, prospective, multicenter studies with standardized follow-up are needed to more definitively assess the impact of surgical platform on disease recurrence.

## Data Availability

The data that support the findings of this study are available from the corresponding author upon reasonable request.
